# The use and limits of ITS data in the analysis of intraspecific variation in *Passiflora* L. (Passifloraceae)

**DOI:** 10.1590/S1415-47572009005000101

**Published:** 2010-03-01

**Authors:** Geraldo Mäder, Priscilla M. Zamberlan, Nelson J. R. Fagundes, Tielli Magnus, Francisco M. Salzano, Sandro L. Bonatto, Loreta B. Freitas

**Affiliations:** 1 Programa de Pós-Graduação em Genética e Biologia Molecular, Departamento de Genética, Instituto de Biociências, Universidade Federal do Rio Grande do Sul, Porto Alegre, RSBrazil; 2 Laboratório de Biologia Genômica e Molecular, Faculdade de Biociências, Pontifícia Universidade Católica do Rio Grande do Sul, Porto Alegre, RSBrazil

**Keywords:** genetic diversity, intraspecific variability, ITS, *Passiflora*, phylogeography

## Abstract

The discovery and characterization of informative intraspecific genetic markers is fundamental for evolutionary and conservation genetics studies. Here, we used nuclear ribosomal ITS sequences to access intraspecific genetic diversity in 23 species of the genus *Passiflora* L. Some degree of variation was detected in 21 of these. The *Passiflora* and *Decaloba* (DC.) Rchb. subgenera showed significant differences in the sizes of the two ITS regions and in GC content, which can be related to reproductive characteristics of species in these subgenera. Furthermore, clear geographical patterns in the spatial distribution of sequence types were identified in six species. The results indicate that ITS may be a useful tool for the evaluation of intraspecific genetic variation in *Passiflora*.

## Introduction

Understanding the distribution of alleles throughout the geographic range of a species is fundamental for molecular evolutionists, allowing inferences about the influence of historical processes in the spatial distribution of particular lineages (Emerson *et al.*, 2001). Phylogeography is a young discipline that represents a bridge linking population genetics, molecular phylogenetics and biogeography, amongst other fields (Avise *et al.*, 1987; Avise, 2000). Phylogenetic methods can be used to construct haplotype trees that would indicate the historical relationships of gene lineages in a population or species. By comparing such trees to the geographical structure of the data one can infer historical patterns of population subdivision, as well as come to an understanding of current distribution of the major genetic clusters in a studied species, which may be useful for conservation purposes (Frankham *et al.*, 2002). Furthermore, well-resolved phylogenies at the species-level or below are necessary to identify the taxa and populations which have undergone recent speciation and those which are currently diverging (Bradshaw *et al.*, 1995; Whittall *et al.*, 2006).

For over two decades, nuclear ribosomal ITS (internal transcribed spacers of the large subunit of ribosomal DNA) has been the most popular molecular marker in the nuclear genome for evolutionary studies in various plant groups (*e.g.* Baldwin *et al.*, 1995; Alvarez and Wendel, 2003; Hughes *et al.*, 2006; Nieto-Feliner and Rosselló, 2007). Even though the use of ITS as a molecular marker might be problematic because of paralogy and other complex evolutionary patterns caused by its highly repetitive nature (Alvarez and Wendel, 2003), the general evolutionary mechanism for this region proposes that most intragenomic copies would share identical sequences as a result of concerted evolution, a homogenizing process that depends on unequal crossing over during meiosis and biased gene conversion (Dover, 1994; Liao, 1999).

Another advantage in using ITS as molecular marker in plants is that it provides an alternative to cpDNA markers, which may inform only part of the history of a species, since cpDNA is usually inherited from only one of the parents (Birky Jr., 2001; Xu, 2005), and thus proposed migration patterns based entirely on cpDNA may be inaccurate when applied to the population as a whole. The ITS region has been successfully used to infer phylogeographic patterns in a wide range of species (Jeandroz *et al.* 1997; Manos *et al.*, 1999; Rodriguez-Lanetty and Hoegh-Guldberg, 2002; Duran *et al.*, 2004; Lorenz-Lemke *et al.*, 2005; Koehler-Santos *et al.*, 2006; Nettel and Dodd, 2007; Yamaji *et al.*, 2007). Furthermore, since the ITS region is flanked by well-conserved rRNA genes, universal primers can be used for widely different plant groups, thereby avoiding the need for developing specific primer sets, as is the case for SSR markers.

*Passiflora* L. is the largest genus in the Passifloraceae, consisting of over 520 species, recently split into four subgenera (Feuillet and MacDougal, 2003). The molecular phylogeny of the genus has been investigated by various authors, and in general is in agreement with the morphological proposition of subgenera classification (Muschner *et al.*, 2003; Yockteng and Nadot, 2004; Hansen *et al.*, 2006). Particularly, Muschner *et al.* (2003) and AK Hansen (PhD. Thesis, University of Texas, 2004) have used ITS to estimate phylogenies for the whole genus. Krosnick and Freudenstein (2005) have used ITS with success to analyze Supersection *Disemma* (subgenus *Decaloba* (DC.) Rchb. Lorenz-Lemke *et al.* (2005) and Koehler-Santos *et al.* (2006) have also assessed intraspecific variability for two *Passiflora* species based on ITS sequences.

In this study, we used ITS sequences to evaluate general patterns of intra and interspecific variation in several species representing all four *Passiflora* subgenera, to test the usefulness of this genetic marker for further phylogeographic studies in this genus. We found general differences in ITS structure between the two most speciose subgenera (*Passiflora* and *Decaloba*), besides identifying geographic associations among lineages in at least six species.

## Materials and Methods

###  Plant material and DNA extraction

**Figure 1:**
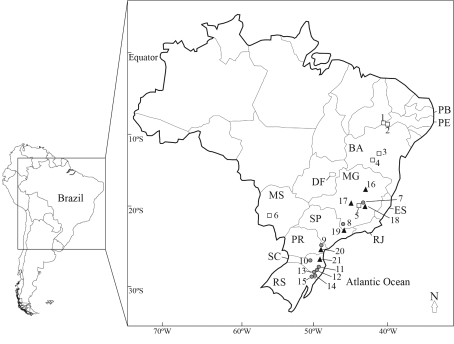
Map of Brazil indicating the origin of samples of the three *Passiflora* species whose preliminary phylogeographical patterns were evaluated. Squares - *P. cincinnata*, circles - *P. organensis,* and triangles - *P. haematostigma*.

Leaf material was obtained from different geographical regions (Table 1). Figure 1 shows collection sites for those species whose preliminary phylogeographical pattern was evaluated. Voucher specimens were deposited in the ICN Herbarium, Botany Department, Biosciences Institute, Federal University of Rio Grande do Sul. Total DNA was extracted from young leaves dried in silica gel, using the method of Roy *et al.* (1992).

###  PCR amplification and sequencing

Internal transcribed spacers (ITS 1 and 2) were amplified using primers and amplification conditions as described by Desfeux and Lejeune (1996). To exclude the presence of low stability templates, 10% dimethyl sulfoxide (DMSO) was used (Buckler IV *et al.*, 1997; Fuertes-Aguilar *et al.*, 1999). PCR products were checked by horizontal electrophoresis in 1% agarose gel stained with GelRed (Biotium). All PCR products were purified using the polyethyleneglycol (PEG) precipitation method (Dunn and Blattner, 1987). The sequencing reaction was performed as described by Sanger *et al.* (1977) using ET terminators kit (GE Healthcare) on a MegaBACE 1000 automatic sequencer (Amersham Biosciences). The quality of the sequences was examined through the Chromas package (available from http://www.technelysium.com.au/ chromas.html). Nucleotide sequences were searched against the GenBank database (http://ncbi.nlm.nih.gov/ BLAST) using BLAST tools (Altschul *et al.*, 1990).

###  Data analysis

GenBank numbers for the obtained sequences are given in Table 1. Forward and reverse reads were analyzed for all sequences. A site was identified as ambiguous when double peaks occurred in the same position in both strands, with the weakest signal reaching at least 25% of the strength of the strongest (Fuertes-Aguilar *et al.*, 1999; Fuertes-Aguilar and Nieto-Feliner, 2003). The term ‘ambiguous' was used instead of ‘heterozygote site', as the origin of ambiguity could not be determined with any degree of certainty, since a variation might represent an actual heterozygote (for which each allele had been inherited from one parent) or only a variation among different ITS copies across the genome. Thus, in all analysis, ambiguous sites were treated as missing data. Sequences were aligned with the PRANK program (Löytynoja and Goldman, 2008). Variable sites, nucleotide and haplotypic diversity, were estimated using the Arlequin 3.11 (Excoffier *et al.*, 2005) software. Relationships among sequences were inferred by means of median-joining networks (Bandelt *et al.*, 1999) under Network version 4.5. Finally, inter and intraspecific distances were estimated with PAUP* version 4.0 (Swofford, 1998), by taking the distance estimated through maximum likelihood, and assuming the evolutionary model suggested under the Aikaike Information Criterion in the Modeltest 3.7 program (Posada and Crandall, 1998). Since the sequences among subgenera were too divergent to be combined in the same alignment, diversity in each subgenus was analyzed separately.

## Results

GC content ranged from 49% to 66% (Table 2 - alignments are available directly from the authors), with an average of 60%, being significantly higher in the subgenus *Passiflora* (63%) than in *Decaloba* (53%) (p < 0.002). ITS1 length varied between 225 bp (*P. eichleriana* Mast.) and 278 bp (*P. misera* HBK), whereas for ITS2, this variation was from 184 bp (*P. sidaefolia* M. Roemer) to 222 bp (*P. caerulea* L.) (Table 2). ITS length was significantly different between the two subgenera for both ITS1 and ITS2 (Table 3). The size of the 5.8S gene (160 bp) was constant in all analyzed species, although a certain intraspecific polymorphism, half of which represented by ambiguous sites, was found in this region in *P. capsularis* L.*,P. edulis* Sims., *P. urnaefolia* Rusby. and *P. villosa* Vell.

**Figure 2:**
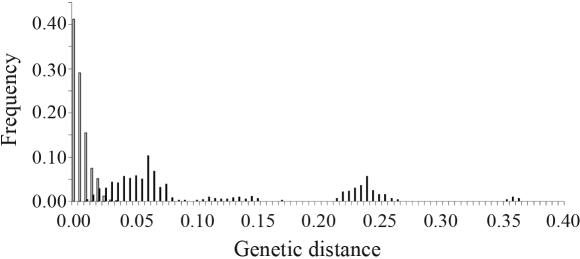
Histogram showing the distribution of frequency values of intra (empty bars) and interspecific (solid bars) genetic distances. Interspecific genetic distances were calculated only comparing species belonging to the same subgenus.

With the exception of *P. pohlii* Mast., in Mart. (subgenus *Decaloba*) and *P. urubiciensis* Cervi (subgenus *Passiflora*), both of which were monomorphic, all species had some degree of intraspecific variability, represented by insertion/deletion (indels) and substitution events, part of which involving ambiguities. When we excluded all ambiguities from the dataset for subsequent analyses we found that three more species become monomorphic (*P. jilekii* Wawra, *P. miersii* Mast. in Mart. and *P. sidaefolia*, all belonging to the subgenus *Passiflora*). The number of nucleotide substitutions was higher than indels, and transitions more numerous than transversions (Table 2). The intraspecific index of nucleotide diversity varied from zero to 0.0227 (Table 2). The highest intraspecific pairwise distance was 0.0447 (*P. foetida* L.). Pairwise interspecific distances between individuals belonging to species from the same subgenus, varied from 0.0128 to 0.0260 in the subgenus *Astrophea* (DC.) Mast. (both between *P. haematostigma* Mart. ex Mast. and *P. rhamnifolia* Mast.), from 0.0049 (*P. tricuspis* Mast. in Mart. *vs.P. organensis* Gard.) to 0.3860 (*P. capsularisvs.P. morifolia* Mast. in Mart.) in *Decaloba* and from zero (*P. miersii* Mast. in Mart. *vs.P. edmundoi* Sacco) to 0.1713 (*P. caeruleavs.P. foetida*) in *Passiflora*. For the subgenus *Deidamioides* (Harms) Killip a single species was studied. The distributions of inter and intraspecific pairwise distances are shown in Figure 2.

As in this study we presented an overview of ITS genetic diversity in *Passiflora* species as a whole, rather than an exhaustive analysis of just a few species, it could be argued that the somewhat limited, and to some extent, unequal sampling of the different species may tend to bias our estimates. To assess the influence of sample size on genetic diversity, we calculated the Spearman correlation coefficient between either sample size or number of sampled populations against two measures of genetic diversity, the number of different sequence types and nucleotide diversity. We observed a significant positive correlation between sample size and both the number of sampled populations and that of different sequence types (rS = 0.601, p = 0.002 and rS = 0.462, p = 0.027, respectively). Nevertheless, when nucleotide diversity was considered, no significant correlation was found with either sample size or the number of sampled localities (rS = 0.070, p = 0.751; and rS = 0.260, p = 0.231; respectively). These results indicate that the values presented here for nucleotide diversity are independent of sampling artifacts, and thus may be viewed as approximations of the level of genetic diversity in these species. However, it is also evident from our data that increased sampling would be required for a better characterization of all sequence types present in a given species.

**Figure 3:**
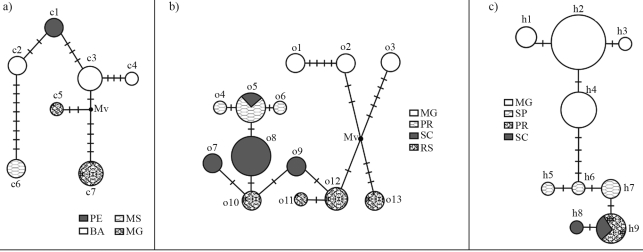
Median-joining networks based on ITS sequence types. Each circle represents a sequence type, their sizes being proportional to respective frequencies. Shading indicates the state in Brazil where each sequence-type was found (according to boxes; key to the abbreviations in Table 4). Transversal bars indicate the number of mutations that differentiate sequence types; Mv: median vector. The differences between the numbers of sequence types indicated in Table 1 for *P.haematostigma* are due to hypervariable sites that were removed for network analysis. 3a - *P. cincinnata*; 3b - *P.organensis*; 3c - *P. haematostigma.* The relationship between sequence types and collection sites is indicated in Table 4.

When considered together with sampling locations, genetic variation for at least six species (*P. haematostigma, P. organensis, P. cincinnata* Mast., *P. edmundoi*, *P. villosa*, and *P. capsularis*) showed some geographic structure as can be observed from the network connecting the different sequence types (Table 4; Figure 3). For *P. cincinnata* (Figure 3a) there was a central core of sequence types located in NE Brazil (BA and PE states), to which the others were connected, the most distantly located population also having the most divergent sequence type. A more complex picture was observed for *P. organensis* (Figure 3b), wherein the most distinct lineages were found at the two extremes of distribution (MG and RS, see Figure1), whereas another group of lineages was arranged in a roughly north/south gradient. A clearer pattern emerged for *P. haematostigma* (Figure 3c), with a clear separation between the sequences from Minas Gerais (MG) and those from other more southern localities, which are arranged in a north/south gradient (SP, PR, SC). Finally, in the three remaining species we also found differences among isolated individuals from different regions albeit with a non-trivial relationship between genetic lineages and geography.

## Discussion

In almost all the species sampled in this study (21 out of 23), a certain degree of genetic variation occurred in the ITS region, even though for some of these only few individuals were sequenced. This is in agreement with other studies, thereby implying that in *Passiflora*, the ITS region seems to be more informative at the populational level than other marker. Lorenz-Lemke *et al.* (2005), when studying 32 individuals of *P. actinia* Hook and 20 individuals of *P. elegans* Mast., and Koehler-Santos *et al.* (2006), studying 32 plants from *P. alata* Curtis, all discovered genetic variation on using ITS but none with cpDNA markers. Lorenz-Lemke *et al.* (2005) found 32 polymorphic sites. Nucleotide diversity was 0.0060 in *P. actinia* and 0.0020 in *P. elegans*, whereas Koehler-Santos *et al.* (2006) detected an ITS nucleotide diversity value of 0.0036 as in *P. alata*.

Patterns of intraspecific variation in *Passiflora* have been also studied by using different markers. More specifically, Fajardo *et al.* (1998) employed RAPD markers and found higher levels of variation in *P. ligularis* Juss. and *P. adenopoda* DC. when compared to *P. edulis* and *P. maliformis* L*.*, whereas Sanchez *et al.* (1999) used cpDNA restriction fragment length polymorphism, and found intraspecific variation in four species (*P. maliformis*, *P. ligularis*, *P. edulis*, and *P. mollissima* (Kunth) Bailey), but no variation in another three (*P*. *caerulea*, *P.* sp*. india*, and *P. adenopoda*). Finally, a morphometric approach undertaken by Plotze *et al.* (2005) found different levels of variation in leaf vein pattern among ten species, with *P. caerulea* being the most diverse. Even though differences among sampled species and markers make a direct comparison of these studies difficult, the emerging overall picture is that intraspecific variation is not evenly distributed among species, and that a common pattern of intraspecific diversity for the nuclear genome, the plastid genome and morphology may be hard to obtain, given the discrepancy of different datasets for the same species (*e.g. P. caerulea*, *P. edulis* and *P. maliformis*). Possibly, this picture reflects the complexities of the evolutionary history of the genus, and indicates that robust patterns would only emerge when different markers are considered together.

In this study we did not take into consideration sites for which two nucleotides could be detected in the same individual. Because there are several ITS repeats and because each individual inherited its genome from both of its parents, it is not possible to know whether sites harboring two different nucleotides are really heterozygous or whether they rather represent ITS sequence variation across different repeats in a single haploid genome. It is therefore impossible to infer meaningful haplotypes based on ITS sequences obtained from PCR amplifications from total DNA. Importantly, because we excluded from our analysis all sites with ambiguous information, the degree of genetic variation in the ITS region in this genus is actually an underestimate. A way of maximizing the detection of genetic variation in ITS sequences would be by cloning all individuals and sequencing multiple clones for each. However, this alternative is not only expensive for phylogeographic and population genetics studies, but might also be subject to criticism because one would be mixing orthologous and paralogous variation in a single analysis (Bailey *et al.*, 2003). AK Hansen (PhD. Thesis, University of Texas, 2004) sequenced ITS clones obtained for 41 *Passiflora* species and observed that in 25% of the studied taxa, various ITS sequences were more related to sequence types in other species. It is not known whether this illustrates the complexity of using paralogue ITS sequences in a single analysis or not, since, according to other studies on the same genus, variation patterns inconsistent with sequence types from a single monophyletic species have never been found (Muschner *et al.*, 2003; Krosnick and Freudenstein, 2005; Lorenz-Lemke *et al.*, 2005).

A comparison of intra and interspecific genetic distances (Figure 2) suggests little overlap between the two levels, with more than 50% of the intraspecific comparisons resulting in values larger than zero, thus stressing the potential of the ITS region as a molecular marker in phylogeographic studies in *Passiflora*. The narrow overlap between intra and interspecific values may be understood in two-ways. First, the intraspecific genetic variation may be inflated if there are unrecognized species within a given taxon name. This may be the case for *P. foetida*, for which 38 varieties are recognized (Killip, 1938), and which showed the highest intraspecific variation (Table 2). A thorough taxonomic revision of this species would therefore considerably reduce the overlap between intra and interspecific genetic variation in the ITS region. Second, interspecific distances are expected to be low between recently diverged species. Thus, we may expect that either recent speciation events or taxonomic confusion would cause an overlap between genetic distances estimated at these two taxonomic levels. This may be of special concern for speciose groups. Moreover, differences represented by indel events are not taken into account when estimating distances, thus probably reducing the overall estimate of interspecific divergence. For example, some individuals from *P. miersii* and *P. edmundoi* are only differentiated by a 2-bp indel in the ITS2 region.

Provided that concerted evolution is often invoked as an explanation for the maintenance of genetic homogeneity among ITS copies, why then does genetic variation in the ITS region seems to be the rule rather than the exception in *Passiflora* species? The time for homogenizing different ITS copies depends on the number of the different copies, on the number of different chromosomes containing nrDNA, and on the crossing-over rate among chromosomes (Quijada *et al.*, 1998), since crossing-over towards telomeres seems to be more common, as previously shown in *Gossypium* L. and *Thinopyrum* A. Löve (Wendel *et al.*, 1995; Li and Zhang, 2002). Melo and Guerra (2003) mapped the 45S ribosomal DNA (including the ITS region) in several *Passiflora* species. In none of the species analyzed (including *P. capsularis, P. cincinnata*, *P. edulis*, *P. haematostigma* and *P. morifolia,*) had nrDNA located in telomeres, this indicating that chromosome location in this genus may be, at least partly, responsible for a slow down in the homogenization of the different ITS copies. This could partially explain, independent of the taxonomy issues, the high values observed for *P. foetida*, which has six nrDNA clusters far from telomeres, in regions of reduced crossing-over.

The differences in ITS1 and ITS2 length and GC content, detected among species in the subgenera *Passiflora* and *Decaloba*, might be related to their significantly different evolutionary rates, as shown by Muschner *et al.* (2003). The latter also ascertained that ITS1 and ITS2 nucleotide diversities were significantly higher in *Decaloba* than in *Passiflora* species. *Decaloba* species are in general self-compatible (Ulmer and MacDougal, 2004), a condition that favors lower intrapopulation and higher interpopulation genetic diversities, as there is less pollen dispersion (Maki *et al.*, 1999). In the present work, no difference was detected when average intraspecific nucleotidic diversities were compared in *Passiflora*. This indicates that, despite the different evolutive rates, ITS intraspecific variability it is not distinct in these subgenera. We consider that the differentiated evolutionary rates in the subgenera *Decaloba and Passiflora* do not represent obstacles to the use of ITS markers for intraspecific populational analysis in *Passiflora* species. However, this question must be handled carefully when dealing with phylogenetic questions in this genus and is worthy of future study.

Even though our study did not aim at detecting fine phylogeographic structure for these species, for at least six species, sampling site location could be related to the observed genetic diversity. Despite the low number of samples, the overall pattern seems to indicate a general latitudinal trend. Of course, detailed phylogeographic studies will be required to reveal accurate geographic patterns, and to infer those evolutionary processes that influenced the distribution of the genetic lineages currently found in these species. It is interesting to note that Lorenz-Lemke *et al.* (2005), also based on ITS sequences, detected similar patterns in *P. actinia* and *P. elegans*. Palynological data indicate two Holocene expansions in the Brazilian Atlantic Rain Forest from the northeast towards south Brazil (Lorscheitter, 1997). Nonetheless, any extensive assessment of whether the history of the genetic lineages within these species indeed reflects concordant colonization routes in the Brazilian Atlantic Rain Forest will await further studies and additional data.

Despite their complex evolution, ITS sequences have high inter and intraspecific variability in *Passiflora* species. This information can be of importance for accessing their evolutionary history and contributing to conservation. At least in some species, a clear geographic structure of sequence types can be identified, with expressive agreement among the different species. As a whole, the results presented here indicate that ITS is a useful tool for evaluating intraspecific variability in *Passiflora* species.

## Figures and Tables

**Table 1:** List of the species studied, their taxonomic position according to the infrageneric classification of Feuillet and MacDougal (2003), and GenBank accession numbers for DNA sequences. The number of individuals examined, the number of collection sites and the geographic origin of the populations are given for each species.

Subgenus	Species	N	Collection sites^a^	Geographic regions^b^	Sequence types^c^	Genbank
*Passiflora*	*P. amethystina*	3	3	RS, SC, SP	3	EU258307 - EU258309
	*P. caerulea*	24	14	RS	4	EU258310 - EU258313
	*P. cinccinata*	7	6	MS, MG, BA, PE	7	EU258352 - EU258358
	*P. edmundoi*	5	4	BA, MG, RJ	5	EU258370 - EU258374
	*P. edulis*	12	8	RS, SC, MG	10	EU258375 - EU258384
	*P. eichleriana*	7	4	RS, SC	3	EU258317 - EU258319
	*P. foetida*	9	6	RS, SC, PE, PB	7	EU258386 - EU258391; EU258394
	*P. jilekii*	5	4	SC	1	EU258320
	*P. miersii*	3	1	SP	1	EU258322
	*P. sidaefolia*	12	2	SP, MG	1	EU258435
	*P. tenuifila*	15	2	RS	8	EU258446 - EU258453
	*P. urubiciensis*	12	11	RS, SC	1	EU258326
	*P. villosa*	6	5	SC, PR, SP, MG	6	EU258392 - EU258393; EU258466 - EU258469
*Decaloba*	*P. capsularis*	42	5	RS, SC, PR, SP, MG	19	EU258327 - EU258345
	*P. misera*	15	5	RS	4	EU258409 - EU258412
	*P. morifolia*	7	5	RS, SP	2	EU258323 - EU258324
	*P. organensis*	17	9	RS, SC, PR, MG	13	EU258414 - EU258426
	*P. pohlii*	5	4	MG, MS	1	EU258325
	*P. tricuspis*	8	4	SP, MS, DF	6	EU258455 - EU258460
	*P. urnaefolia*	9	4	RS, SP, MG	4	EU258462 - EU258465
*Astrophea*	*P. haematostigma*	19	6	SC, PR, SP, MG	9	EU258395 - EU258403
	*P. rhamnifolia*	12	5	MG	6	EU258427 - EU258429; EU258432 - EU258434
*Deidamioides*	*P. ovalis*	18	7	ES, BA	11	EU258359 - EU258369

^a^The collection sites for *P. cinccinataP. organensis* and *P. haematostigma* are shown in Figure 1. ^b^Abbreviations refer to Brazilian states: RS: Rio Grande do Sul; SC: Santa Catarina; PR: Paraná; SP: São Paulo; RJ: Rio de Janeiro; ES: Espírito Santo; MG: Minas Gerais; MS: Mato Grosso do Sul; DF: Distrito Federal; BA: Bahia; PE: Pernambuco. ^c^Sequence types found for the whole region (ITS1 + 5.8S + ITS2).

**Table 2:** *Passiflora* taxa considered and sequence characteristics of the data. All polymorphisms (transitions, transversions and indels) refer to individual alignments for each species. Values within brackets correspond to the number of polymorphic sites for which two nucleotides were detected. Values within parentheses refer to standard deviation.

Subgenera and species	ITS1					5.8 S				ITS2				%GC	Nucleotide diversity	Sequence diversity
	Length	Ts	Tv	Indels		Ts	Tv	Indels		Length	Ts	Tv	Indels			
*Passiflora*																
*P. amethystina*	227	0 [0]	0 [0]	0		0 [0]	0 [0]	0		215	3 [0]	2 [0]	1	64	0.0050 (0.0044)	1.0000 (0.2722)
*P. caerulea*	227	3 [1]	0 [0]	0		0 [0]	0 [0]	0		222	1 [1]	0 [0]	1	63	0.0010 (0.0009)	0.5000 (0.0665)
*P. cinccinata*	233	5 [1]	6 [0]	2		0 [0]	0 [0]	0		218	4 [1]	4 [1]	0	63	0.0104 (0.0059)	0.9231 (0.0367)
*P. edmundoi*	226	5 [1]	3 [1]	0		0 [0]	0 [0]	0		218	2 [0]	3 [0]	2	64	0.0092 (0.0054)	0.8889 (0.0596)
*P. edulis*	227	6 [2]	5 [1]	0		1 [0]	0 [0]	0		219	3 [1]	2 [1]	2	64	0.0060 (0.0035)	0.9130 (0.0350)
*P. eichleriana*	225	1 [0]	1 [0]	0		0 [0]	0 [0]	0		220	1 [1]	3 [0]	0	64	0.0024 (0.0019)	0.2857 (0.1964)
*P. foetida*	230	26 [15]	7 [3]	8		0 [0]	0 [0]	0		214	14 [3]	6 [1]	14	60	0.0227 (0.0119)	0.8627 (0.0573)
*P. jilekii*	226	1 [1]	0 [0]	0		0 [0]	0 [0]	0		219	0 [0]	0 [0]	0	63	0	0
*P. miersii*	226	0 [0]	0 [0]	0		0 [0]	0 [0]	0		220	1 [1]	0 [0]	0	62	0	0
*P. sidaefolia*	226	4 [4]	5 [5]	0		0 [0]	0 [0]	0		184	2 [2]	1 [1]	0	66	0	0
*P. tenuifila*	226	10 [3]	3 [1]	0		0 [0]	0 [0]	0		218	0 [0]	4 [1]	0	63	0.0067 (0.0038)	0.8535 (0.0246)
*P. urubiciensis*	226	0 [0]	0 [0]	0		0 [0]	0 [0]	0		220	0 [0]	0 [0]	0	61	0	0
*P. villosa*	228	7 [1]	5 [2]	4		0 [0]	1 [1]	0		216	4 [0]	5 [1]	2	63	0.0168 (0.0093)	0.9091 (0.0459)
*Decaloba*																
*P. capsularis*	271	11 [4]	0 [0]	0		3 [1]	0 [0]	0		203	11 [1]	3 [0]	6	55	0.0037 (0.0023)	0.9168 (0.0156)
*P. misera*	278	3 [1]	1 [0]	0		0 [0]	0 [0]	0		198	6 [1]	0 [0]	0	53	0.0014 (0.0011)	0.3873 (0.0969)
*P. morifolia*	275	4 [0]	1 [0]	0		0 [0]	0 [0]	0		198	1 [0]	2 [0]	0	54	0.0033 (0.0022)	0.2637 (0.1360)
*P. organensis*	277	10 [5]	9 [1]	0		0 [0]	0 [0]	0		199	2 [2]	0 [0]	0	54	0.0045 (0.0027)	0.9269 (0.0228)
*P. pohlii*	276	0 [0]	0 [0]	0		0 [0]	0 [0]	0		199	0 [0]	0 [0]	0	52	0	0
*P. tricuspis*	277	4 [3]	0 [0]	0		0 [0]	0 [0]	0		201	2 [2]	0 [0]	0	49	0.0008 (0.0008)	0.8667 (0.0467)
*P. urnaefolia*	276	0 [0]	2 [1]	0		1[1]	0 [0]	0		200	2 [1]	0 [0]	0	53	0.0009 (0.0009)	0.6536 (0.0982)
*Astrophea*																
*P. haematostigma*	269	5 [5]	0 [0]	0		0 [0]	0 [0]	0		208	3 [1]	5 [1]	4	63	0.0047 (0.0028)	0.7568 (0.0663)
*P. rhamnifolia*	269	3 [1]	4 [1]	0		0 [0]	0 [0]	0		204	1 [1]	0 [0]	0	63	0.0022 (0.0016)	0.8116 (0.0465)
*Deidamioides*																
*P. ovalis*	236	2 [2]	2 [2]	1		0 [0]	0 [0]	0		212	6 [3]	4 [0]	1	64	0.0048 (0.0028)	0.8635 (0.0433)

**Table 3:** *Passiflora* subgenera average characteristics. Values within parentheses refer to the range of variation or standard deviation.

Subgenus	Species number	% GC	ITS1 length	ITS2 length	Ambiguous sites number	Nucleotide diversity	Sequence diversity
*Passiflora*	13	63* (60-66)	227* (225-233)	216* (184-222)	4 (0-22)	0.0062 (0.0036)	0.5489 (0.0611)
*Decaloba*	7	53* (49-54)	276* (271-278)	200* (198-203)	3 (0-8)	0.0021 (0.0014)	0.5736 (0.0595)
*Astrophea*	2	63	269	206 (204-208)	5 (3-7)	0.0035 (0.0022)	0.7842 (0.0564)
*Deidamioides*	1	64	236	212	7	0.0048 (0.0028)	0.8635 (0.0433)

*Statistically significant values (p < 0.002).

**Table 4:** Collection points of *Passiflora* species considered in phylogeographic analyses.

Species	Sampling sites	Geographic coordinates	Sequence types
*P. cincinnata*	1- Casa Nova/BA	09° 09'43” S/40° 58'15” W	c2
	2- Petrolina/PE	09° 23'55” S/40° 30'03” W	c1
	3- Abaíra/BA	13° 14'59” S/41° 39'49” W	c3,c4
	4- Caetité/BA	14° 07'36” S/42° 26'05” W	c3
	5- Brumadinho/MG	20° 08'36” S/44° 11'59” W	c7
	6- Jardim/MS	21° 28'49” S/56° 07'53” W	c6
*P. organensis*	7- Caeté/MG	19° 52'56” S/43° 40'12” W	o3
	8- Munhoz/MG	22° 36'31” S/46° 21'41” W	o1, o2
	9- Quatro Barras/PR	25° 20'16” S/48° 54'49” W	o4, o5, o6
	10- Petrolândia/SC	27° 35'29” S/49° 44'14” W	o5, o8, o9
	11-Bom Jardim da Serra/SC	28° 22'14” S/49° 34'41” W	o8
	12- Timbé do Sul/SC	28° 49'42” S/49° 50'30” W	o8
	13- Praia Grande/SC	29° 07'25” S/49° 58'29” W	o7
	14- Três Cachoeiras/RS	29° 26'53” S/49° 55'37” W	o10
	15- Maquiné/RS	29° 34'36” S/50° 17'35” W	o11, o12, o13
*P. haematostigma*	16- São Gonçalo do Rio Preto/MG	18° 00'15” S/43° 23'27” W	h2, h4
	17- Araújos/MG	19° 53'59” S/45° 15'21” W	h2
	18- Itabirito/MG	20° 22'01” S/43° 39'40” W	h1, h2, h3, h4
	19- Biritiba/SP	23° 39'04” S/46° 08'17” W	h5, h6, h7
	20- Guaratuba/PR	25° 52'58” S/48° 34'29” W	h9
	21- Rancho Queimado/SC	27° 40'59” S/49° 01'59” W	h8, h9
